# A Role for the Nonsense-Mediated mRNA Decay Pathway in Maintaining Genome Stability in *Caenorhabditis elegans*

**DOI:** 10.1534/genetics.117.203414

**Published:** 2017-06-20

**Authors:** Víctor González-Huici, Bin Wang, Anton Gartner

**Affiliations:** School of Life Sciences, Centre for Gene Regulation and Expression, University of Dundee, DD1 5EH, UK

**Keywords:** ionizing radiation, double-strand-break repair, nonsense-mediated mRNA decay, transcription-replication interface, mitosis

## Abstract

Ionizing radiation (IR) is commonly used in cancer therapy and is a main source of DNA double-strand breaks (DSBs), one of the most toxic forms of DNA damage. We have used *Caenorhabditis elegans* as an invertebrate model to identify novel factors required for repair of DNA damage inflicted by IR. We have performed an unbiased genetic screen, finding that *smg-1* mutations confer strong hyper-sensitivity to IR. SMG-1 is a phosphoinositide-3 kinase (PI3K) involved in mediating nonsense-mediated mRNA decay (NMD) of transcripts containing premature stop codons and related to the ATM and ATR kinases which are at the apex of DNA damage signaling pathways. Hyper-sensitivity to IR also occurs when other genes mediating NMD are mutated. The hyper-sensitivity to bleomycin, a drug known to induce DSBs, further supports that NMD pathway mutants are defective in DSB repair. Hyper-sensitivity was not observed upon treatment with alkylating agents or UV irradiation. We show that SMG-1 mainly acts in mitotically dividing germ cells, and during late embryonic and larval development. Based on epistasis experiments, SMG-1 does not appear to act in any of the three major pathways known to mend DNA DSBs, namely homologous recombination (HR), nonhomologous end-joining (NHEJ), and microhomology-mediated end-joining (MMEJ). We speculate that SMG-1 kinase activity could be activated following DNA damage to phosphorylate specific DNA repair proteins and/or that NMD inactivation may lead to aberrant mRNAs leading to synthesis of malfunctioning DNA repair proteins.

EFFICIENT repair of DNA damage is important for cell survival and for preventing the accumulation of mutations, which can lead to major diseases, such as cancer, premature aging, and neurodegeneration. Paradoxically, cancer treatment often involves the use of genotoxic agents to kill cancer cells, and one of the most effective therapies is ionizing radiation (IR). Arguably, the most toxic DNA lesions inflicted by IR are DNA double-strand breaks (DSBs), and the effectiveness of radiotherapy is strongly influenced by the capacity of cells to repair DSBs. Typically, two main mechanisms have been described to intervene in DSB repair, homologous recombination (HR) and classical nonhomologous end-joining (NHEJ) (reviewed in Kass and Jasin 2010; Ceccaldi *et al.* 2016). HR involves the switch to an undamaged sister template to copy the information lost at the lesion site, and thus tends to be error-free and predominantly used during S and G2 phases of the cell cycle. NHEJ involves ligation of DNA ends with minimal processing. This pathway is considered moderately error-prone, as small 1- to 4-nt deletions are usually generated. More recently, the “alternative” or “microhomology-mediated” end-joining (MMEJ), an error-prone pathway which generates insertions and deletions, has been implicated as a third, major DSB repair modality (Ceccaldi *et al.* 2015; Mateos-Gomez *et al.* 2015). This error-prone mechanism, which requires resection at the break site, depends on DNA polymerase Theta (θ) and prevents large deletions in regions of the *Caenorhabditis elegans* genome, which are hard to replicate through G4 structures (Koole *et al.* 2014; Roerink *et al.* 2014; van Schendel *et al.* 2015).

*C. elegans* is the simplest animal model system to study DNA damage responses, and fundamental contributions to the DNA repair field have been made in the last two decades. Following an unbiased genetic screen to find new factors that protect from IR we have identified SMG-1. SMG-1 is a PI3K kinase, present in higher eukaryotes but not in yeast (for reviews see Schoenberg and Maquat 2012; Metze *et al.* 2013; Schweingruber *et al.* 2013; Lykke-Andersen and Jensen 2015; Hug *et al.* 2016). Nonsense-mediated mRNA decay (NMD) acts immediately after mRNAs are exported at the first pioneer round of translation. During this translation, exon junction complexes formed in the nucleus to mark splice sites are normally removed from mRNAs. However, when an exon junction complex persists downstream of a stop codon, this complex links up with the NMD machinery being bridged by the conserved UPF2/SMG3 and UPF3/SMG4 factors. The most important reaction mediated by the captured NMD complex is the activation of the SMG1 kinase, whose activity is normally kept at bay by SMG8 and SMG9. SMG1 phosphorylation leads to the activation of the UPF1/SMG2 helicase. This leads to mRNA unwinding and protein removal, followed by mRNA cleavage via the SMG6 nuclease and SMG5- and SMG6-dependent recruitment of mRNA decapping and deadenylation factors, all together facilitating the degradation of mRNAs with premature stop codons.

NMD is considered to help adjusting transcriptomes and proteomes to varying physiological conditions (Ramani *et al.* 2009; Schoenberg and Maquat 2012; Lykke-Andersen and Jensen 2015). NMD plays a fundamental role in aggravating human genetic diseases due to mRNA degradation, where a premature stop codon does not lead to a full loss of function (reviewed in Miller and Pearce 2014). NMD has also been involved in stress response and modulation of the unfolded protein response (UPR) threshold (Karam *et al.* 2013, 2015) as well as in the inflammatory immune response (Mino *et al.* 2015). NMD is often inhibited in tumors, as a consequence of stresses, like starvation, hypoxia, or infection (Gardner 2010; Karam *et al.* 2013) which negatively regulate the pioneer round of translation via phosphorylation of the translation initiation factor eIF2α (Gardner 2008; Wang *et al.* 2011a,b). A well-known example of a tumor protective effect of NMD is conferred by the degradation of mutant brca1 mRNAs that encode for truncated dominant-negative forms of this protein (Perrin-Vidoz *et al.* 2002). The human SMG1 kinase is a target of the ATM/ATR PI3 kinases, which act at the apex of DNA damage response pathways (Matsuoka *et al.* 2007). Smg1 knockout mice are early embryonic lethal (Brumbaugh *et al.* 2004) and heterozygous animals are reported to be cancer-prone (Roberts *et al.* 2013). Partial depletion of human SMG1 by RNAi induced modest IR sensitivity (Gubanova *et al.* 2012, 2013).

There is evidence for roles of SMG1 independent of its NMD function. hSMG1 has a reported role in the nucleus in processing the long noncoding telomeric repeat containing RNA (TERRA) needed for regulating telomerase activity (Azzalin *et al.* 2007), while a *C. elegans smg-1* mutant was described to show oxidative stress resistance and extended lifespan in a CEP-1/p53-dependent manner (Masse *et al.* 2008). *C. elegans* NMD mutants do not have any overt developmental defect, but global changes in transcriptome of NMD- defective worms have been reported (Ramani *et al.* 2009). Here, we report the identification of a new *smg-1* allele leading to a D1789N point mutation in the PIK domain of SMG-1, which confers IR sensitivity. The same phenotype occurs in previously reported *smg-1* mutations and in a large panel of mutations affecting other components of the NMD-pathways. The level of IR sensitivity is comparable to previously reported DSB repair mutants. Epistasis analysis shows a synergistic effect with the mutants affecting the main DSB repair pathways, namely HR, NHEJ, and MMEJ, thus suggesting that SMG-1 is involved in a parallel pathway. In summary, we genetically define a major role of the NMD pathway in maintaining genome stability in the *C. elegans* organismal model.

## Materials and Methods

### *C. elegans* strains and maintenance

Worms were maintained at 20° on *E. coli* OP-50 seeded NGM agar plates, as described previously (Brenner 1974). Alleles are all described in Wormbase. The N2 Bristol reference line TG1813 is used in the Gartner laboratory as the wild-type reference strain. All mutant strains were backcrossed six times to TG1813. Strains are listed in Supplemental Material, Table S1.

### EMS mutagenesis screening and identification of IR-sensitive mutants

Wild-type worms (P0) were mutagenized with 25 mM ethyl methanesulfonate (EMS) for 4 hr following the standard methodology. F2 generation single L4 stage worms were transferred to each well in 96-well tissue culture plates and cultured in OP50-containing (OD_600_ = 0.1) liquid medium for 3 days. Then the grown worm mixtures were filtered with 0.45-μm pore size Millipore Nylon Net filters (11 μm NY11) fitted in the 96-well plates to get the L1 stage worms, which were divided into two parts. One plate was irradiated with a ^137^Cs source (60 Gy), and L1 worms were subsequently cultured to select IR-hyper-sensitive mutants. The second plate is kept to recover radiation-sensitive worms. For identification of the mutations responsible for the IR-hyper-sensitivity phenotype the strains were backcrossed three times against the Bristol reference strain and then crossed once with a Hawaiian strain. F2 worms showing the IR-sensitivity phenotype were pooled and sequenced. Using the Galaxy platform for mapping of Hawaiian variants (https://usegalaxy.org/u/gm2123/p/cloudmap) (Minevich *et al.* 2012), we identified a region in chromosome 1 displaying nearly 100% Bristol SNPs, which includes the *smg-1* *(tg2855)* mutation.

### Radiation/genotoxin sensitivity assays

For IR and UV L1 assays, early L1 larvae were transferred to seeded NGM plates and irradiated at the indicated doses as previously described (Bailly *et al.* 2010; Craig *et al.* 2012). For bleomycin assays, L1s were incubated in M9 buffer containing OP50 and the indicated concentration of bleomycin for 2 hr at 20° before centrifuging and plating worms. Worms were allowed to reach young adult stage and distributed in three plates containing three worms each. After 12 hr, worms were removed and total egg number counted.

For assays performed on young adults, worms were irradiated as described for L1s, transferred to fresh plates, and allowed to lay eggs for 12 hr at the indicated time intervals (Bailly *et al.* 2010; Craig *et al.* 2012). For methyl methanesulfonate (MMS), aflatoxin B1, and aristolochic acid intoxication, worms were incubated for 16 hr at 20° in OP50-containing M9 buffer, centrifuged, left to recover for 24 hr in NGM-seeded plates, then three worms/plate were transferred to three plates and allowed to lay eggs for 6 hr.

For late embryo IR-sensitivity assays, used to assay for DNA end-joining defects, we followed the procedure as previously described (Clejan *et al.* 2006), irradiating at the indicated doses and scoring for the extent of the growth defect (GRO phenotype) 48 hr later. Uncoordinated (UNC) and ruptured (RUP) phenotypes were scored 96 hr after irradiation.

### RAD-51 and DAPI staining

For the chromosome fractionation assay, bacteria were washed off intact worms with M9 buffer, then M9 was replaced with 100 ng/ml DAPI in 100% ethanol and allowed to evaporate for ∼30 min. Worms were rehydrated in M9 for 1 hr, then transferred to a drop of Vectashield mounting solution in a coverslip, slides mounted, and the coverslip sealed with nail polish. Diakinetic chromosomes, corresponding to oocytes in position −1, −2, and −3, were visualized using a DeltaVision wide-field microscope and images captured with a Coolsnap HQ camera. For RAD-51 staining, worms were dissected and germlines released and fixed prior to immunostaining. Germlines-containing slides were incubated with rabbit anti-RAD51 antibodies (1/800) overnight (MacQueen *et al.* 2002; Alpi *et al.* 2003), washed, and incubated with a secondary Alexa568 anti-rabbit antibody diluted 1/750 in PBS containing 1 μg/ml DAPI for 2 hr. Slides were washed, worms mounted with Vectashield, and coverslip edges sealed. Images were captured as described above using the TRITC and DAPI channels.

### Apoptosis assay

Following irradiation, apoptotic corpses were visualized by Normarski microscopy, as previously described (Craig *et al.* 2012).

### Data availability

All strains are available upon request, and genotypes are described in Table S1.

## Results

### SMG-1 is involved in IR resistance

**Figure 1:**
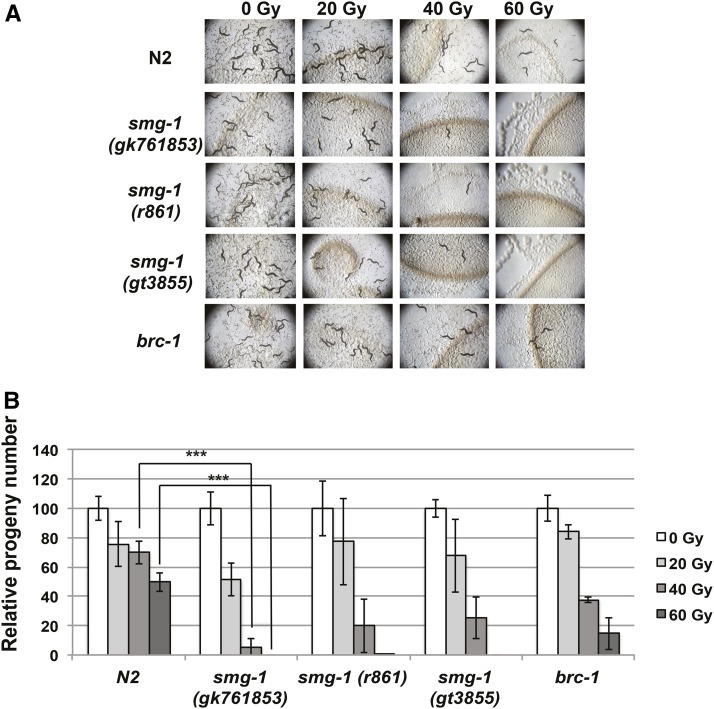
*smg-1* mutants are hyper-sensitive to IR. Early L1 larvae of N2 (wild type), *smg-1* (*gk761853*), *smg-1* (*r861*), *smg-1* (*gt3855*), and *brc-1* (*tm1145*) were subjected to the indicated amounts of radiation, and allowed to develop to young adult stage and lay eggs. After 12 hr worms were removed and eggs counted. (A) Images of the plates 6 days after egg-laying, evidencing the impaired fertility of *smg-1* mutants after irradiation. (B) Number of laid eggs normalized to that of the nonirradiated specimen for each strain. Three plates with three worms each were scored for each strain and condition. Error bars indicate SD. Asterisks indicate the level of significance as standard: * *P* = 0.01–0.05, ** *P* = 0.001–0.01, ***P < 0.001.

We conducted an unbiased genetic screen to identify new genes involved in IR response. EMS was selected as a mutagen and the mutagenized F2 progeny were exposed to IR. Hyper-sensitivity was assessed by loss of fertility, and identification of the mutation responsible carried out by positional cloning, facilitated by SNP mapping and Next Generation Sequencing, as described in the *Materials and Methods*. Here, we focus on one of the most sensitive mutations we identified: *smg-1* (*tg3855*) that leads to a D1789N change in the *smg-1* gene (Figure S1A). Aspartic Acid 1789 is conserved in all PI3 kinases and embedded in a highly conserved tract of 42 aa (33/42 identity, 35/42 similarity to hsSMG1) within the PIK domain of the protein. We confirmed that hyper-sensitivity to IR is associated with *smg-1* deficiency using two further *smg-1* alleles, previously shown to be null alleles: *r861*, the molecular nature of which has not been described (Hodgkin *et al.* 1989; Grimson *et al.* 2004), and importantly also the truncation allele *gk761853*, which causes a premature stop codon at position 289 and thus does not contain the PI3 kinase domain (Figure S1B). The latter allele was generated as part of the. *C. elegans* million mutation project (Thompson *et al.* 2013) and like the (*tg3855*) allele was backcrossed six times against N2 to eliminate unlined mutations. To systematically characterize the IR sensitivity of these *smg-1* alleles, we treated L1 larvae with IR and allowed them to develop into adult-stage worms. Six days after irradiation, proliferation of F1 progeny, resulting from L1 stage P0 generation worms, was scored, clearly showing a reduced number of progeny (Figure 1A). This assay is generally accepted to measure IR sensitivity in mitotically proliferating germ cells (Bailly *et al.* 2010; Craig *et al.* 2012). At the early L1 stage, worm germ cells are composed of only two germ cells, which expand to ∼1000 cells in each of the two germ lines. Thus, reduced germ cell proliferation is a measure of radiation sensitivity, leading to sterility in extreme cases, while the number and viability of progeny sired from those germ cells is reduced, when lower IR doses are applied (Bailly *et al.* 2010; Craig *et al.* 2012). A mutation in the *brc-1* gene, the *C. elegans* ortholog of the mammalian *brca1* HR repair gene, served as a positive control (Boulton *et al.* 2004) (Figure 1A). Results were quantified by scoring for the number of viable embryos (Figure 1B). We found that all three *smg-1* alleles are significantly more sensitive to IR than wild type at 40 and 60 Gy (*P* < 0.001), where complete sterility was observed. In summary, our data confirm that *smg-1* mutations lead to hyper-sensitivity to IR and that the strength of this phenotype is at least as strong as observed upon deleting canonical genes involved in response to DSB damage, such as *brc-1* (Figure 1) and *lig-4* (Figure 4C and Figure 5A), the latter being involved in NHEJ.

### *smg-1* radio-sensitivity is largely due to hyper-sensitivity of mitotically dividing cells

**Figure 2:**
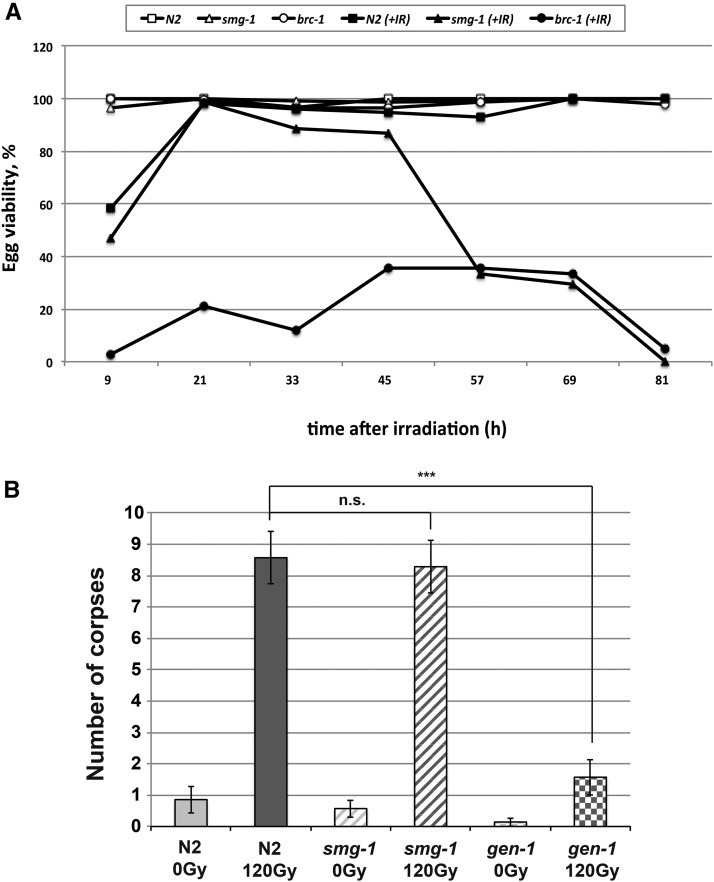
(A) Embryonic viability at different times postirradiation. Young adults of N2, *smg-1* (*gk761853*), and *brc-1* (*tm 1145*) were irradiated (60 Gy), and embryonic viability, calculated as the percentage of hatched eggs for both irradiated and nonirradiated strains, was scored in 12-hr intervals (0–9, 9–21, 21–33, 33–45, 45–57, 57–69, 69–81 hr), at the end of which worms were transferred to fresh plates. Six plates were scored for each strain, time, and condition. (B) DNA-damage-induced germ cell apoptosis is not affected by *smg-1* mutants. N2 (wild type), *smg-1* (*gk761853*), and *gen-1* (*tm2940*) were irradiated at 120 Gy and the number of apoptotic corpses scored after 24 hr. Error bars show SD. Level of significance indicated as *** *P* < 0.001 and n.s., not significant, *P* > 0.05.

We wanted to determine whether SMG-1 requirement to withstand IR was associated with germ cells going through mitosis, meiosis, or both. To address this, we analyzed worms irradiated at the young adult stage. At this stage, germ cell expansion and differentiation is already well advanced. Germ cells are differentiated into a distal compartment where mitotic proliferation continuously occurs while later a large number of germ cells reside in the meiotic pachytene stage, a stage where meiotic chromosomes synapse and no proliferation occurs. When such worms are treated and the relative survival of embryos that are laid after ∼24 hr is scored, these typically derive from pachytene cells; if embryonic survival is scored after 48 or more hours, these are typically derived from mitotic germ cells (Bailly *et al.* 2010; Craig *et al.* 2012). If the very first embryos, laid after ca. 12 hr, are scored these are typically derived from very late stage meiotic cells, in the diplotene and diakinesis stage. We found that *smg-1* mutants behaved like the wild-type when scored after 24 hr, but strong sensitivity occurred when scoring was done after 48 hr or longer (Figure 2A). This was in sharp contrast to *brc-1* worms, which were IR-sensitive irrespective of when they were scored. The assay was performed with the *smg-1* (*gk761853*) allele and the same results were obtained with the *smg-1* (*r861*) allele (data not shown). We previously established that persistent DNA damage and recombination failure in pachytene cells leads to the activation of a p53-dependent checkpoint leading to germ cell apoptosis. We thus counted the number of apoptotic corpses 24 hr after treating wild-type and *smg-1* mutant worms with 120 Gy of IR. We found that apoptosis was induced as in wild type. Thus, the DNA damage checkpoint, in contrast to *gen-1* mutations is not compromised, and increased apoptosis does not occur (Figure 2B). No differences were spotted either when apoptosis was scored 48 hr postirradiation (data not shown). Therefore, SMG-1 does not seem to be required for correct apoptosis following DNA damage. In summary, our combined data strongly suggest radiation sensitivity associated with *smg-1* deficiency due to mitotically dividing germ cells.

### *smg-1* is hyper-sensitive to the DSB-producing drug bleomycin

**Figure 3:**
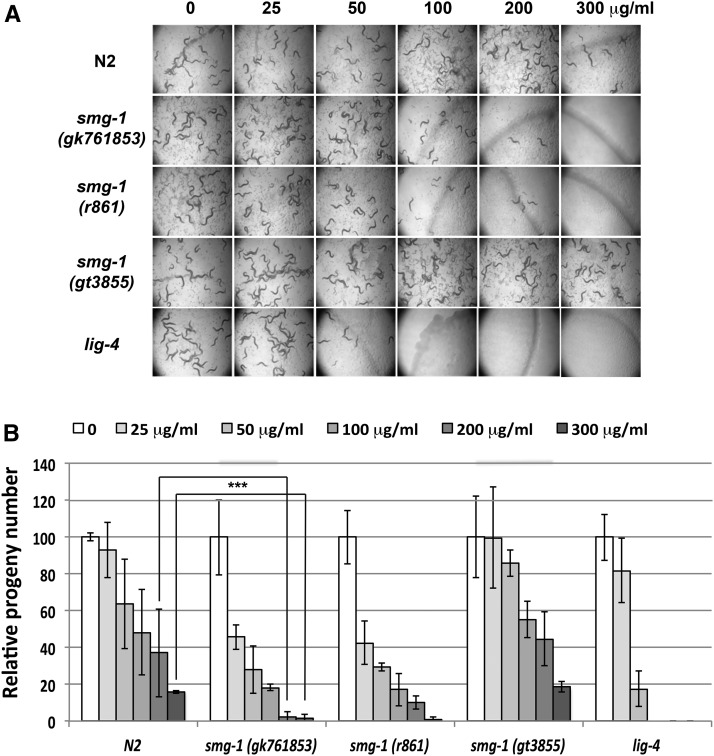
Sensitivity of *smg-1* mutants to bleomycin: Early stage, nonstarved L1 larvae of N2, *smg-1* (*gk761853*), *smg-1* (*r861*), *smg-1* (*gt3855*), and *lig-4* (*ok716*) were incubated in M9 buffer for 2 hr with the indicated amounts of bleomycin, transferred to drug-free plates, and allowed to develop to young adult stage and lay eggs. For each strain and condition, three plates, each containing three young adults, were scored. After 12 hr, worms were removed and eggs counted. (A) Images of the plates 6 days after egg-laying. (B) Number of laid eggs normalized to that of the nonirradiated specimen for each strain. Error bars indicate SD. Asterisks indicate the level of significance as standard: * *P* = 0.01–0.05, ** *P* = 0.001–0.01, *** *P* < 0.001.

Even if DSBs are the most toxic lesion induced by IR, direct or free radical-mediated ionization can produce other types of DNA damage, like single-strand breaks (SSBs) or base damage, as well as oxidation of proteins and membranes. Therefore, we decided to assay for the sensitivity of *smg-1* mutants to bleomycin, a radiomimetic drug known to induce DSBs (Povirk *et al.* 1989). Following 2 hr incubation of early L1 stage animals in bleomycin, we plated larvae on drug-free NMG plates and controlled developmental delay 48 hr later. Unlike IR, bleomycin induces a significant developmental delay, which is exacerbated in *smg-1* mutants (Figure S2). At 300 μg/ml bleomycin and 48 hr after exposure, N2 has only 2% adults as compared to 100% in the untreated sample. Most worms are at the L3–L4 stage, with 20% not developing beyond the L1–L2 stage. In *smg-1* mutant worms, the developmental delay is further accentuated, the majority of worms not progressing beyond the L2 stage (Figure S2). We next analyzed the ability of young adults treated with lower doses of bleomycin to produce progeny following bleomycin exposure at the L1 stage. We prepared three plates, each with three worms, per strain and dose, allowed 12 hr for egg-laying, counted them, and allowed progeny to self-propagate. Figure 3A shows the images of the plates 6 days after egg-laying, while Figure 3B shows the number of eggs laid. Consistent with the developmental delay data, *smg-1* (*gk761853*) and *smg-1* (*r861*) show impaired fertility already at 25 μg/ml bleomycin (*P* = 0.004 and 0.005, respectively), while such an effect requires 100 μg/ml in N2 (*P* = 0.012). At 200 μg/ml they are nearly sterile, and no progeny at all can be detected at 300 μg/ml, when N2 still shows a significant, albeit reduced, propagation capacity. Interestingly, *smg-1* (*gt3855*) behaves as wild-type, with a significant reduction only at 100 μg/ml (*P* = 0.002). This could be due to a separation of function, as a result of a residual activity conferred by this allele or possibly also due to a genetically linked suppressor mutation.

### SMG-1 acts in parallel to the main DSB repair pathways

**Figure 4:**
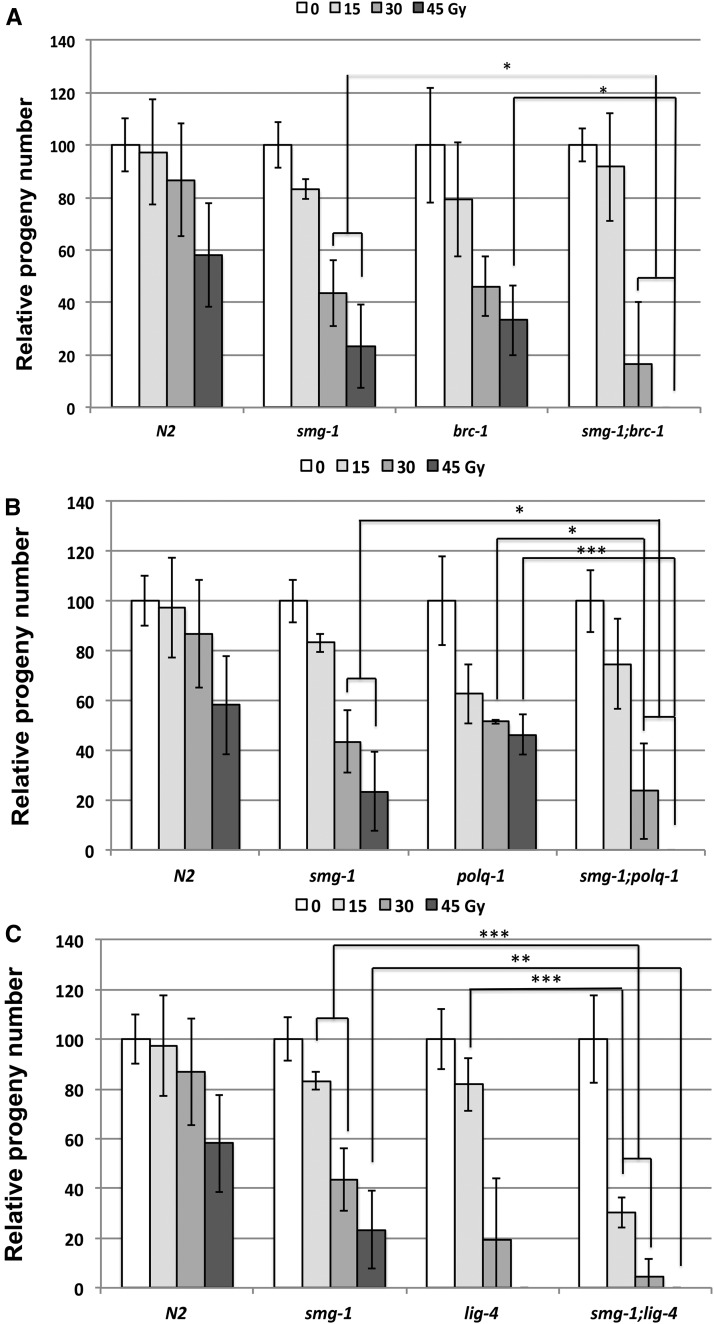
Epistasis analysis of *smg-1* with the main DSB repair pathways: Early L1 larvae of N2, *smg-1 (gk761853)*, plus (A) *brc-1 (tm1145)* and *smg-1 (gk761853)*; *brc-1 (tm1145)*, (B) *polq-1 (tm2026)* and* smg-1 (gk761853)*; *polq-1 (tm2026)*, and (C) *lig-4 (ok716)* and *smg-1 (gk761853)*; *lig-4 (ok716)* were subjected to the indicated amounts of radiation, allowed to develop to young adult stage and lay eggs. After 12 hours worms were removed and eggs counted. For each strain and condition a minimum of 3 plates, each containing 3 young adults, were scored. Error bars indicate SD. Asterisks indicate the level of significance as standard: * *P* = 0.01-0.05, ** *P* = 0.001-0.01, ***P < 0.001.

Three main pathways have been described to repair DSBs: homologous recombination (HR), nonhomologous end-joining (NHEJ), and microhomology-mediated end-joining (MMEJ). We carried out an epistasis analysis designing double mutants of *smg-1* (*gk761853*) either with *brc-1* (HR), *lig-4* (NHEJ), or *polq-1* (MMEJ) and subjecting them to IR (15, 30, and 45 Gy) during early L1 stage. In all cases the double mutants were significantly more sensitive than the single mutants (Figure 4, A and B). The synergistic effect of *smg-1*; *lig-4* double mutants is even more pronounced. Already at 15 Gy, *smg-1*; *lig-4* is much more sensitive than *smg-1* or *lig-4* (*P* < 0.001) (Figure 4C). Altogether, these results strongly suggest that SMG-1 does not function as a canonical factor of any of the three pathways.

We then carried out a similar epistasis analysis using bleomycin as described for Figure 3. Double mutant *smg-1*; *brc-1* produced significantly less progeny than the single mutants at 50 and 100 μg/ml bleomycin (Figure S3A). The same was observed for *smg-1*; *polq-1* and the respective single mutants after 50 and 100 μg/ml bleomycin treatment (Figure S3B). These results further support the above observations using IR and the conclusion of SMG-1 acting independently of the main DSB repair pathways.

**Figure 5:**
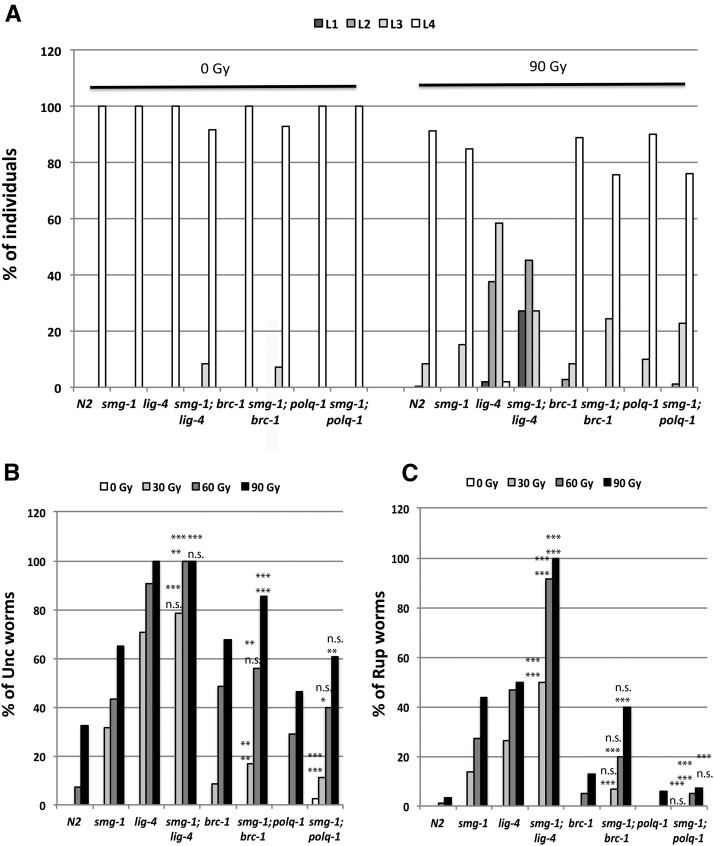
‘Epistasis analysis of smg-1 with the main DSB repair pathways: Late embryos of N2, *smg-1 (gk761853)*, *lig-4 (ok716)*, *smg-1 (gk761853)*; *lig-4 (ok716)*, *brc-1 (tm1145)*, *smg-1 (gk761853)*; *brc-1 (tm1145)*, *polq-1(tm2026)*, and *smg-1 (gk761853)*; *polq-1 (tm2026)* were subjected to the indicated amounts of radiation and allowed to develop. 3 phenotypes, indicative of genotoxicity, were scored. (A) GRO phenotype: 48 h later the percentage of worms at each of the 4 larval stages was scored. (B) UNC phenotype: 96 h later the percentage of worms moving uncoordinatedly was scored. (C) RUP phenotype: 96 h later we scored the percentage of ruptured worms, meaning extrusion of the gut through the vulva. Asterisks indicate the level of significance of the differences observed in double mutants with respect to the respective singles as standard: n.s *P* > 0.05, * *P* = 0.01-0.05, ** *P* = 0.001-0.01, ***P < 0.001. Top line indicates significance respect to smg-1 and bottom to *lig-4*, *brc-1* or *polq-1*.

Finally, we decided to analyze these epistatic relationships following irradiation of late-stage embryos. It has been previously established that during this stage, DSB repair relies mainly on NHEJ, and sensitivity can be assessed by a developmental delay (Clejan *et al.* 2006). Without IR, the growth of any single mutant was not delayed and a slight delay occurred in *smg-1*; *lig-4* and *smg-1*; *polq-1* double mutants. As expected, *lig-4* mutants showed a strong developmental delay after treatment with 90 Gy, and this was strongly accentuated in *smg-1*; *lig-4* lines (Figure 5A). When late-stage *lig-4* embryos were treated with IR, developmental phenotypes arose due to problems with the proliferation of somatic cells in the larval stages (Clejan *et al.* 2006). Many neurons are only born in larvae, and hence an uncoordinated (UNC) phenotype where worms do not move at all or not ordinarily can form. Also, several cell divisions are required for the proper formation of the vulva, and if the vulva does not properly form, worms rupture, with a germ line protruding through the defective vulva (RUP phenotype) (O’Connell *et al.* 1998) (Figure 5, B and C). Using these assays, *smg-1* shows a significant increase in sensitivity as compared to wild type (at 60 Gy, 43 *vs.* 7% UNC and 27 *vs.* 1% RUP), the strongest phenotypes as expected being observed in *lig-4* mutants. Especially upon scoring for the RUP phenotype a synergistic increase in the double mutant is observed. This synergistic effect is much less evident in *smg-1*; *brc-1*, significant only for the UNC phenotype at 90 Gy (85 *vs.* 65% for *smg-1* and 68% for *brc-1*) (Figure 5, B and C). All in all, these data show that the SMG-1 also contributes to genome stability during somatic development, and that it synergizes with the end-joining pathway.

### *smg-1* sensitivity to other genotoxins

**Figure 6:**
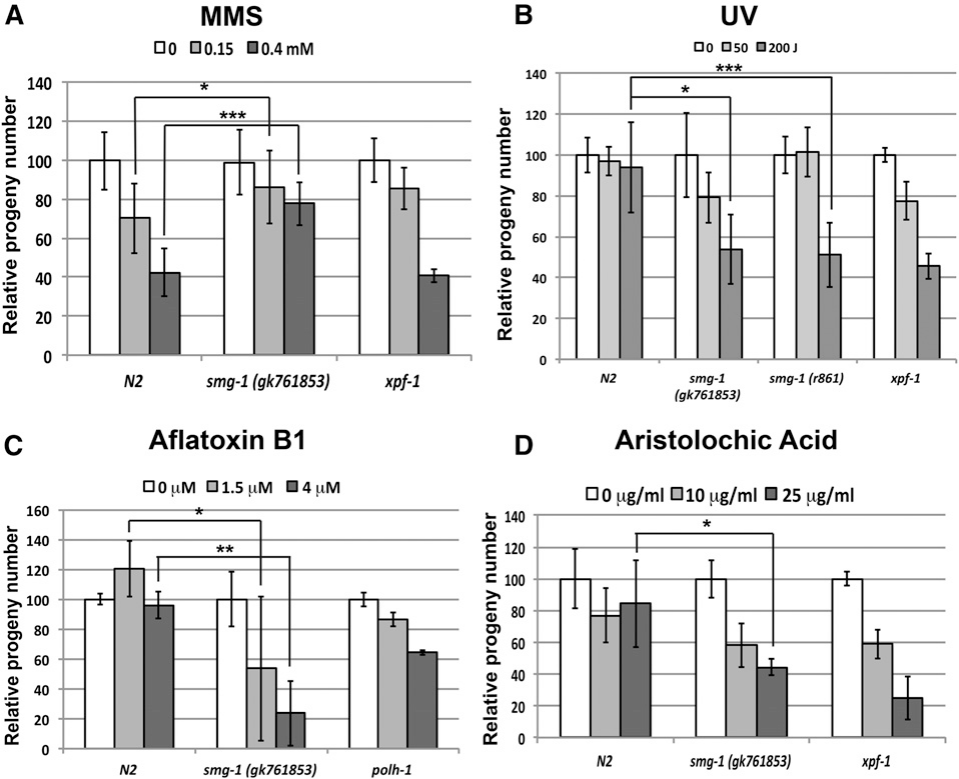
Sensitivity of *smg-1* mutants to various genotoxins. (A) Young adults of N2, *smg-1* (*gk761853*), and *xpf-1* (*tm2842*) were exposed to the indicated MMS doses for 16 hr, allowed to recover for 24 hr and then the number of laid eggs in a 6-hr period assessed and normalized to the untreated conditions. A minimum of three plates with three worms each per strain and dose were used. (B) Early L1 larvae of N2, *smg-1* (*gk761853*), and *smg-1* (*r861*) were subjected to the indicated amounts of UV irradiation and allowed to develop to young adult stage and lay eggs. After 12 hr, worms were removed, eggs counted, and normalized to the untreated specimen value. At least three plates with three worms each per strain and dose were used. (C) As in A, but intoxicating worms with aflatoxin B1 and using *polh-1* (*ok3317*) as a positive control. (D) As in A, but intoxicating worms with aristolochic acid. Error bars indicate SD. Asterisks indicate the level of significance as standard: * *P* = 0.01–0.05, ** *P* = 0.001–0.01, ***P < 0.001.

We then decided to investigate whether SMG-1 is required for the response to further DNA-damaging agents and therefore intoxicated *smg-1* mutants with a battery of genotoxins that induce various DNA lesions. Methyl methanesulfonate (Figure 6A) is a drug which damages DNA by methylating bases at several positions, the most toxic intermediates being O6-methylguanine and N3-methyladenine (Fu *et al.* 2012). Interestingly, we found that *smg-1* mutant worms are partially resistant against MMS. Sensitivity to UV light, which produces mainly pyrimidine dimers and intrastrand cross-links, is increased in *smg-1* mutants (Figure 6B) at the highest dose of 200 J/m. We found a much more pronounced effect of *smg-1* when we used genotoxins that damage DNA via addition of bulky adducts, like aflatoxin (Figure 6C), a mycotoxin that is a major health concern in many parts of the world associated with fungal food contamination and aristolochic acid. The latter compound is a phytotoxin linked to the outbreak of rare types of kidney cancers in the Balkan area where *Aristolochia* spp. are endemic, as well as upon the consumption of herbal teas where *Aristolochia* is included (Stiborova *et al.* 2016). Treatment with both agents leads to reduced survival of *smg-1* mutant worms to an extent that exceeds the reduction of viability observed in *xpf-1* and *polh-1* worms (Figure 6, C and D). These results suggest that SMG-1 may play additional roles in response to DNA damage not directly linked to DSB generation.

### IR sensitivity of mutants in the NMD pathway

**Figure 7:**
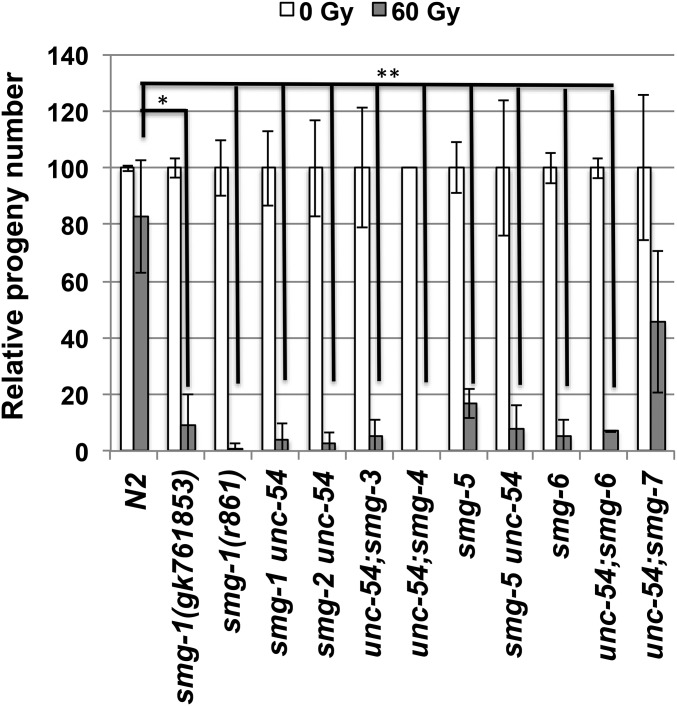
IR sensitivity of other NMD mutants**:** Early L1 larvae of N2, *smg-1(gk761853)*, *smg-1(r861)*, *smg-1(r904) unc-54(r293)*, *smg-2(r908) unc-54(r293)*, *unc-54 (r293)*; *smg-3 (r930)*, *unc-54 (r293)*; *smg-4 (r1169)*, *smg-5 (r860)*, *smg-5 (r860) unc-54 (r293)*, *smg-6 (ok1794)*, *unc-54 (r293)*; *smg-6 (r1217)* and *unc-54 (r293)*; *smg-7 (r1197)* were subjected to the indicated amounts of radiation, allowed to develop to young adult stage and lay eggs. After 12 hours worms were removed and eggs counted. The number of laid eggs normalized to that of the non-irradiated specimen for each strain is shown. Three plates with 3 worms each were scored for strain and condition. Error bars indicate SD. Asterisks indicate the level of significance as standard: * *P* = 0.01-0.05, ** *P* = 0.001-0.01, ***P < 0.001.

We then tried to establish whether the IR hyper-sensitivity of *smg-1* is due to a SMG-1 function outwith the NMD pathway or whether IR hyper-sensitivity is generally associated with NMD defects. We therefore repeated the IR sensitivity assay described in Figure 1 using a battery of mutants carrying mutations in different genes involved in the NMD pathway (Figure 7). Many of these carry an *unc-54* mutation, which is suppressed by NMD deficiency and does not affect IR sensitivity. Scoring upon treatment with 60 Gy we found that all *smg* mutants analyzed showed reduced survival to an extent comparable to *smg-1*. Interestingly, the IR sensitivity of *unc-54*; *smg-7* is only modestly increased (*P* = 0.087) (Figure 7), in line with the partial rescue of the *unc-54* phenotype in this double mutant (data not shown) and consistent with previous data that NMD is only partially impaired in *smg-7* mutants (Metze *et al.* 2013). Given that increased IR sensitivity tallies with NMD deficiency, these results strongly suggest that SMG-1 function in NMD is at least partially responsible for its role in conferring resistance to IR.

### *smg-1* mutation increases RAD-51 foci number after irradiation but does not induce chromosome fragmentation

Finally, we decided to directly investigate DSB induction in *smg-1* worms. To achieve this we chose to stain for the RAD-51 recombinase, which marks resected DSBs engaging in recombinational repair, with RAD-51 driving strand invasion. RAD-51 foci are readily detected locating to DNA damage sites following irradiation (Alpi *et al.* 2003; Colaiacovo *et al.* 2003). We irradiated young adults with 120 Gy, extracted germlines by dissecting worms at different times postirradiation, stained with DAPI and anti-Rad-51 antibodies, and analyzed mitotically dividing germ cells (Figure S4). While RAD-51 foci are virtually nonexistent in the absence of irradiation, the *smg-1* mutant exhibits a significantly higher number of RAD-51 foci as compared to wild-type after irradiation with 120 Gy 2, 6, 24, and 48 hr after irradiation (Figure S4A, File S1, File S2, File S3, File S4, File S5, File S6, File S7, File S8, File S9, File S10, and File S11). These results are in line with an increased number of IR-induced DSBs in *smg-1* mutants.

A further assay to directly measure repair of IR-induced DSBs is by interrogating for chromosome fragmentation in diakinesis oocytes 48 hr after subjecting young adults to 60 Gy. As shown in Figure 2, embryonic viability plummets 45 hr after irradiation, such that this timing allows to visualize oocytes that were mitotically dividing germ cells at the time of irradiation. As previously shown (Bailly *et al.* 2010), such treatment leads to chromosome fragmentation in *gen-1* Holliday Junction resolvase mutants. In wild-type, repair occurred and six DAPI-stained bodies indicative of normally differentiated bivalent chromosomes could be observed (Figure S5, A and B) (Bailly *et al.* 2010). SMG-1 behaved like wild type. These data suggest that while there might be a modest increase in DSBs as measured by the number of RAD-51 foci, the repair defect associated with *smg-1* in contrast to *gen-1* does not lead to the fragmentation of meiotic chromosomes (Figure S5, A and B).

## Discussion

Ionizing radiation is not only a public health concern, but also a major therapeutic tool, which is currently used in ∼50% of all cancer patients (Baskar *et al.* 2012). Therefore, a better understanding of how cells mend DNA lesions induced by IR is important to improve the efficiency of tumor treatment. In this paper, we report the identification of *C. elegans* SMG-1, the apical effector of the NMD pathway as a key factor protecting *C. elegans* from DNA damage. Indeed, our data are consistent with the entire NMD pathway being required for genome maintenance.

IR can damage DNA directly or, by inducing the ionization of molecules, particularly water (Lehnert 2007). This process generates free radicals, such as hydroxyl or superoxide reactive oxygen species, which can interact with DNA, leading to base damage and SSBs and DSBs. Base damage and SSBs can be repaired by base excision repair and ligation and pose relatively minor hazards compared to DSBs, the most mutagenic and carcinogenic lesion induced by IR (Von Sonntag 1987; Ward 1998). We hypothesize that a defective NMD pathway leads to an increased number of DSBs in response to IR. This hypothesis is supported by the hyper-sensitivity of *smg-1* worms to bleomycin, as well as by the increased number of RAD-51 foci, a surrogate for DNA DSBs, we observe in *smg-1* worms treated with IR.

We consider three main not mutually exclusive hypotheses to explain the mechanism by which SMG-1 contributes to the DSB repair. First, NMD could affect the expression of DNA repair proteins. SMG-1 is absolutely required for NMD in *C. elegans* (Hodgkin *et al.* 1989), and the fact that we observe the same IR sensitivity phenotype in all NMD mutants of the NMD pathway we examined supports this hypothesis. This hypothesis is in line with recent observations made in the budding yeast system where it was shown that the levels of RAD55, RAD51, RAD54, and RAD57 recombination proteins were regulated by NMD (Janke *et al.* 2016). These authors did not report sensitivity to IR, but found that affected mutants are resistant to MMS. Indeed, their findings are in line with the decreased MMS sensitivity we observed. It is estimated that, across species, 3–20% of mRNAs from protein-coding genes are regulated, directly or indirectly, by NMD (Mendell *et al.* 2004; Wittmann *et al.* 2006; Maquat and Gong 2009); a 20% value is estimated for *C. elegans* (Ramani *et al.* 2009). This means that NMD could affect various DNA repair pathways differently by regulating the expression of multiple DNA repair proteins.

Second, the NMD pathway could directly impinge on DNA repair processes, possibly also independently of its function in NMD. For instance, SMG1, which is targeted by the ATR kinase, could coordinate DNA repair enzymes by DNA-damage-induced phosphorylation (Matsuoka *et al.* 2007). While NMD typically occurs in the cytoplasm, human SMG1 has a reported role in the nucleus in processing the long noncoding telomeric repeat, containing RNA (TERRA) needed for the regulation of telomerase activity (Azzalin *et al.* 2007). Short noncoding RNAs (sncRNAs) are produced at the site of DSBs and contribute to their efficient repair (Francia *et al.* 2012; Wei *et al.* 2012). We postulate that SMG-1 could affect the expression of noncoding RNAs, such as sncRNAs. It is known that long noncoding RNAs (lncRNAs) are regulated by the NMD pathway (Wery *et al.* 2016). UPF1, the human homolog of *C. elegans* SMG-2, is an RNA- and DNA-dependent 5′-3 helicase activated by hSMG1 phosphorylation (He and Jacobson 2015). Intriguingly, this helicase, the mutation of which also leads to IR sensitivity in *C. elegans* (Figure 4), seems to have nuclear roles as its RNAi depletion in mammalian cells leads to S-phase arrest, and the protein is further enriched on chromatin upon IR (Azzalin and Lingner 2006). Thus, SMG-2/UPF1 regulated by SMG-1 could regulate DNA repair processes by processing an RNA, DNA, or a RNA/DNA hybrid structure functioning as a DNA repair intermediate.

Finally, we consider a role at the interface between RNA metabolism and repair. There is emerging evidence of important links between transcription and genome instability. R-loops are hybrid structures formed when an emerging mRNA anneals to the template DNA strand, thereby displacing the complementary DNA strand. Failure to process and remove R-loops results in hyper-recombination and genome instability (Aguilera and Garcia-Muse 2012), potentially because R-loops can cause replisome stalling, which may in turn cause DSBs. Alternatively, such structures could prevent DSB processing. While NMD is occurring in the cytoplasm, NMD-dependent mRNA degradation is triggered within a minute of mRNA export, concomitant with the pioneer round of translation. Thus, the failure to process these early transcripts could affect the number or nature of R-loops in the nucleus.

In summary, we found that SMG-1 and the NMD pathway play an important role to protect cells against IR. NMD mutants are equally sensitive to IR as canonical DSB repair mutants, but it remains to be determined how NMD mechanistically affects DSB repair. Nevertheless, our results indicate that the NMD pathway could be a possible target for radio-sensitization in cancer treatment.

## Supplementary Material

Supplemental material is available online at www.genetics.org/lookup/suppl/doi:10.1534/genetics.117.203414/-/DC1.
