# Supplementary material for: A Role for the Nonsense-Mediated mRNA Decay Pathway in Maintaining Genome Stability in Caenorhabditis elegans
Source: Genetics. 2017 Jun 20;206(4):1853–64. doi: 10.1534/genetics.117.203414 (PMC5560793; doi:10.1534/genetics.117.203414)
Supplement: Supplementary file 11 [file 1853FileS11.docx]

SUPPLEMENTARY FIGURES LEGENDS

FIGURE S1. Identification of *smg-1* mutation conferring IR hypersensitivity: (A) Following sequencing of pooled F1s from Bristol x Hawaii cross we used the Galaxy platform to process data. We determined a region of chromosome I to be enriched to near 100% in Bristol SNPs, making it the best match to contain the mutation responsible for the phenotype (top). We located a D/N mutation in the *smg-1* gene (lower panel) in a tract where virtually all sequence reads corresponded to the N2 reference strain. (B) Schematic domain structure of the SMG-1 protein. The positions of the (*gt3855*) mutation we identified and the (*gk761853*) premature stop mutations are indicated.

FIGURE S2. Developmental delay following L1 intoxication with bleomycin: N2 (wild-type), *smg-1* (*gk761853*), *smg-1* (*r861*), and *smg-1* (*gt3855*) were intoxicated with several doses of bleomycin during early L1 stage, plated and allowed to develop for 48 h. At this point the number of individuals at each developmental stage was scored. Only results for the highest dose (300 μg/ml), together with untreated control, are shown. A minimum of 100 worms for each strain and condition were scored.

FIGURE S3. Epistasis analysis of *smg-1* with mutants affecting (A) HR and (B) MMEJ for bleomycin sensitivity: Early L1 larvae of the indicated genotypes were incubated in the presence of the indicated doses of bleomycin for 2h, plated, allowed to reach young adult stage and lay eggs. After 12 hours worms were removed, eggs counted and normalized to the untreated specimen value. At least 3 plates with 3 worms each per strain and dose were used. Strains used were N2, *smg-1 (gk761853)*, *brc-1 (tm1145)*, *smg-1 (gk761853)*; *brc-1 (tm1145)* and (B) *polq-1 (tm2026)* and *smg-1 (gk761853)*; *polq-1 (tm2026).*

FIGURE S4. RAD-51 foci following *smg-1 (gk761853)* irradiation: RAD-51 foci were scored at the indicated times post IR with 120 Gy. Corresponding movies are shown in Supp. Movies 1-10. The average numbers of RAD-51 foci per mitotic nuclei are shown. The 20 mitotic germ cell nuclei closed to the germline distal tip, were scored. Number of nuclei scored was at least 40. Error bars indicate s.e.m.

FIGURE S5. Chromosome fractionation assay: (A) Representative images of DAPI-stained bodies in diakinetic oocytes in *smg-1 (gk761853)* germlines 48 h after irradiation with 60 Gy. *gen-1 (tm2940)* was used as a positive fractionation control. In normal conditions 6 bivalents are visible. (B) Average number of DAPI-stained bodies in diakinetic oocytes. A minimum of 24 oocytes were scored. Error bars indicate SD.

TABLE S1. Strains used in this study: All the mutants were isogenic to N2 wild-type strain.

SUPPLEMENTARY MOVIES. RAD-51 foci (red) in mitotic germ cells following 120 Gy irradiation: DAPI staining is blue. Movie S1. N2, non-irradiated; movie S2. *smg-1 (gk761853)*, non-irradiated; movie S3. N2, 2h post-IR; movie S4. *smg-1 (gk761853)*, 2h post-IR; movie S5. N2, 6h post-IR; movie S6. *smg-1 (gk761853)* , 6h post-IR; movie S7. N2, 24h post-IR; movie S8. *smg-1 (gk761853)* , 24h post-IR; movie S9. N2, 48h post-IR; movie S10. *smg-1 (gk761853)* , 48h post-IR.
