# Supplementary material for: A Role for the Nonsense-Mediated mRNA Decay Pathway in Maintaining Genome Stability in Caenorhabditis elegans
Source: Genetics. 2017 Jun 20;206(4):1853–64. doi: 10.1534/genetics.117.203414 (PMC5560793; doi:10.1534/genetics.117.203414)

FIGURE S1

A

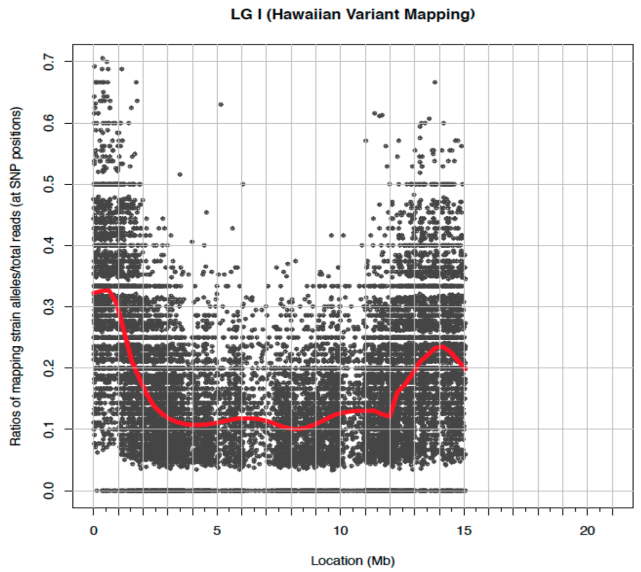

# Chr	Position	Refer	Change	Change	Homoz	Quality	Coverage	Gene_name	Bio_type	Effect	old_AA/new_AA	Old_codon/New_codon
I	2379644	C	T	SNP	Hom	435.13	12	sydn-1	protein_codi	NON_SYNONYMOUS_CODING	M/I	atG/atA
I	5042625	*	#NAME?	DEL	Hom	424.9	10	C46H11.7	protein_codi	CODON_DELETION	T/-	acc/-
I	6627884	C	T	SNP	Hom	473.86	13	D2092.10	protein_codi	NON_SYNONYMOUS_CODING	S/N	aGt/aAt
I	6801829	*	#NAME?	INS	Hom	689.09	13	E02D9.1	protein_codi	SPLICE_SITE_ACCEPTOR		
I	6903858	C	T	SNP	Hom	664.99	19	smg-1	protein_codi	NON_SYNONYMOUS_CODING	D/N	Gat/Aat
I	6903858	C	T	SNP	Hom	664.99	19	smg-1	protein_codi	NON_SYNONYMOUS_CODING	D/N	Gat/Aat
I	7301404	C	G	SNP	Hom	732.53	20	ttx-7	protein_codi	NON_SYNONYMOUS_CODING	G/A	gGc/gCc
I	7301404	C	G	SNP	Hom	732.53	20	ttx-7	protein_codi	NON_SYNONYMOUS_CODING	G/A	gGc/gCc
I	10113280	*	#NAME?	DEL	Hom	1004.58	27	Y106G6D.3	protein_codi	FRAME_SHIFT: Y106G6D.3		
I	11145688	*	#NAME?	INS	Hom	582.05	14	ZK39.9	protein_codi	FRAME_SHIFT: ZK39.9		
I	13101700	*	#NAME?	DEL	Hom	230.37	12	Y26D4A.21	protein_codi	FRAME_SHIFT: Y26D4A.21		
I	14349946	*	+C	INS	Hom	902.92	21	Y105E8A.2	protein_codi	SPLICE_SITE_DONOR		

B

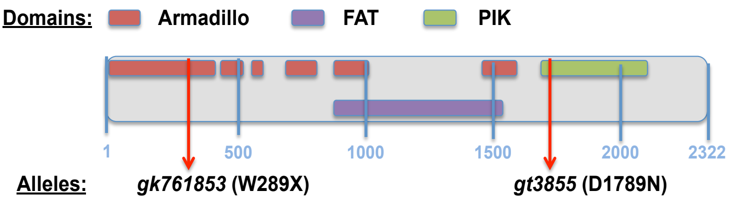

Supplement: Supplementary file 12 [file 1853FigureS1.pdf]
