# Supplementary figures and images for: A Role for the Nonsense-Mediated mRNA Decay Pathway in Maintaining Genome Stability in Caenorhabditis elegans
Source: Genetics. 2017 Jun 20;206(4):1853–64. doi: 10.1534/genetics.117.203414 (PMC5560793; doi:10.1534/genetics.117.203414)

FIGURE S2

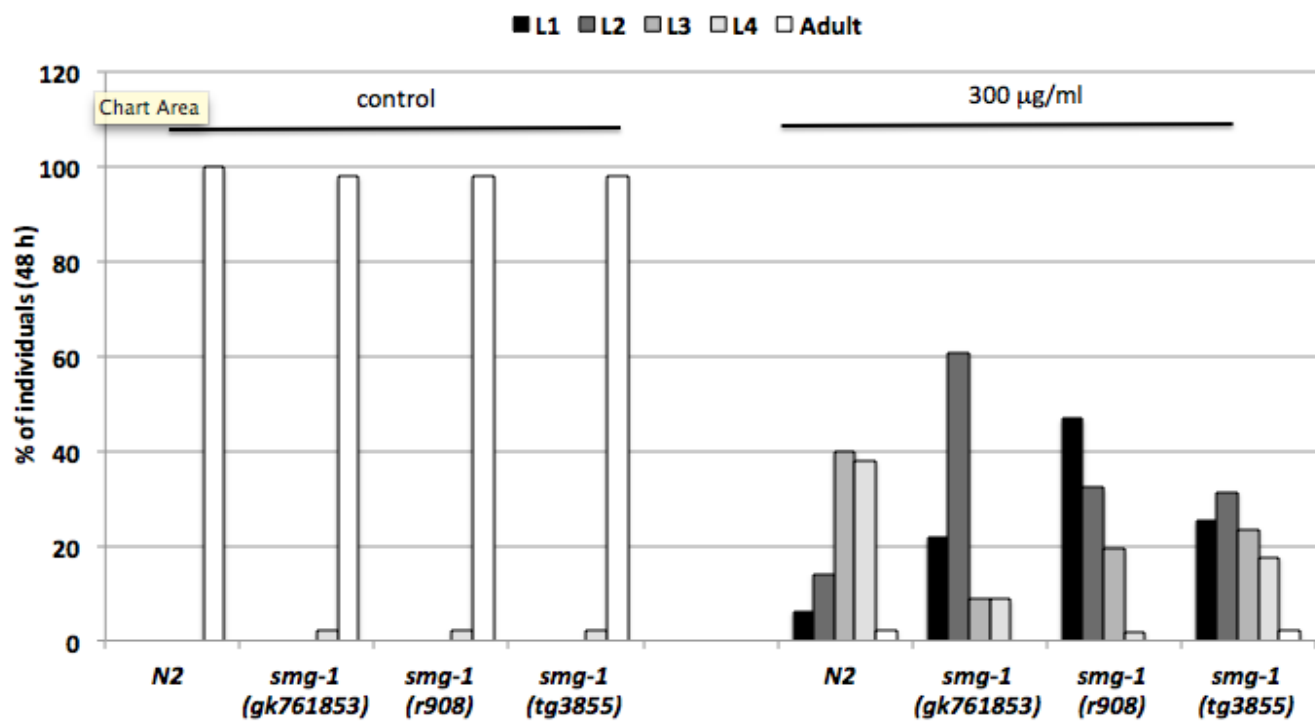

Supplement: Supplementary file 13 [file 1853FigureS2.pdf]

FIGURE S3

A

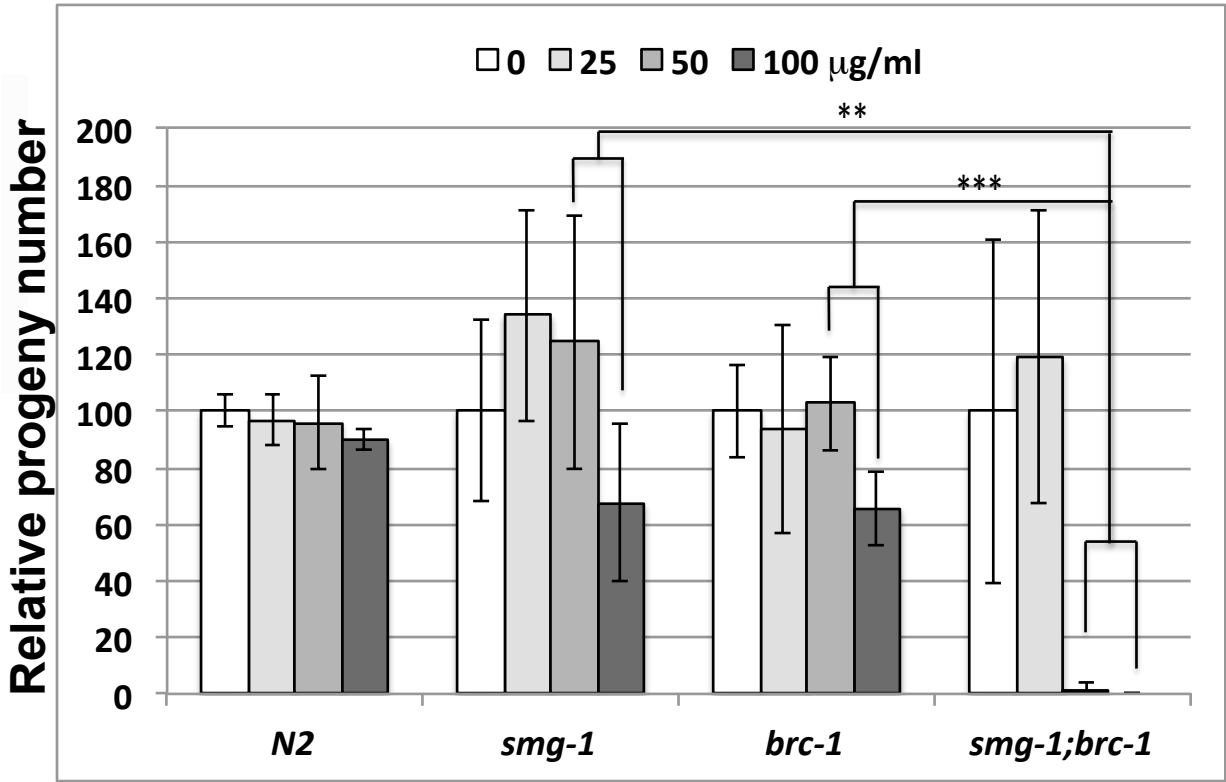

B

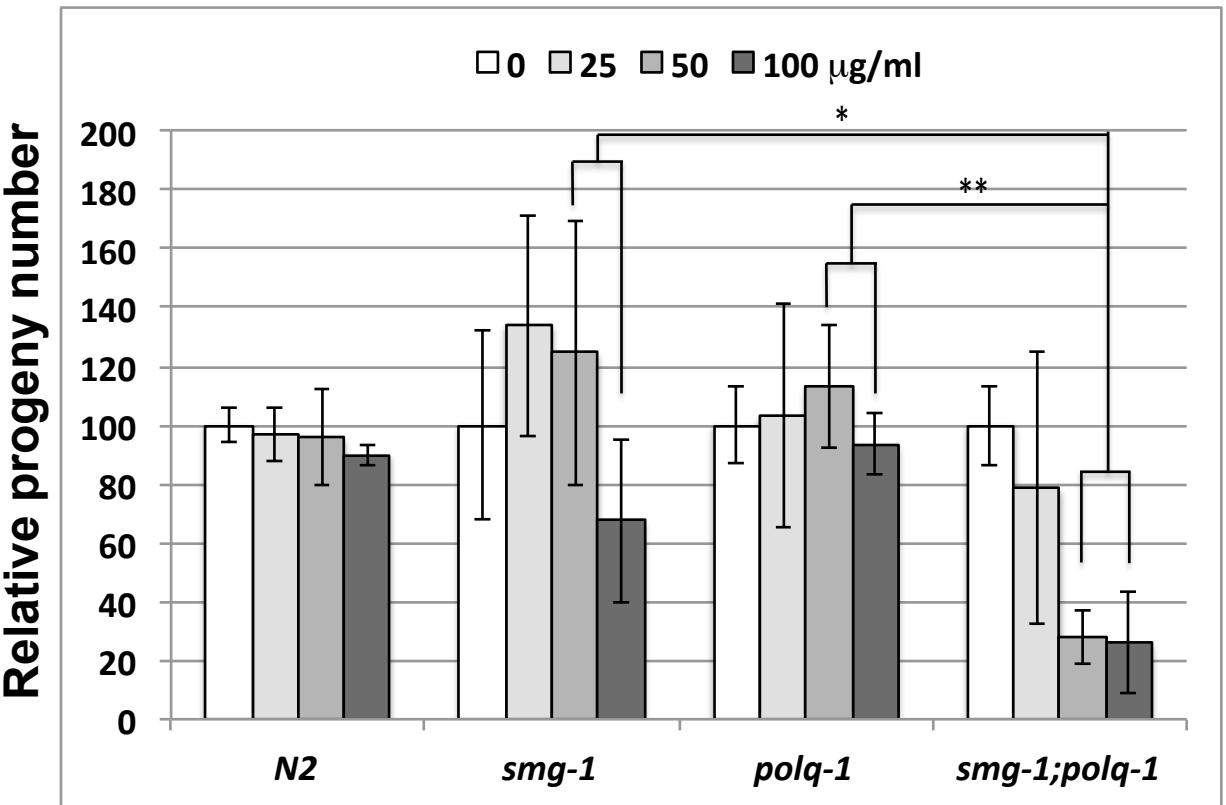

Supplement: Supplementary file 14 [file 1853FigureS3.pdf]

FIGURE S4

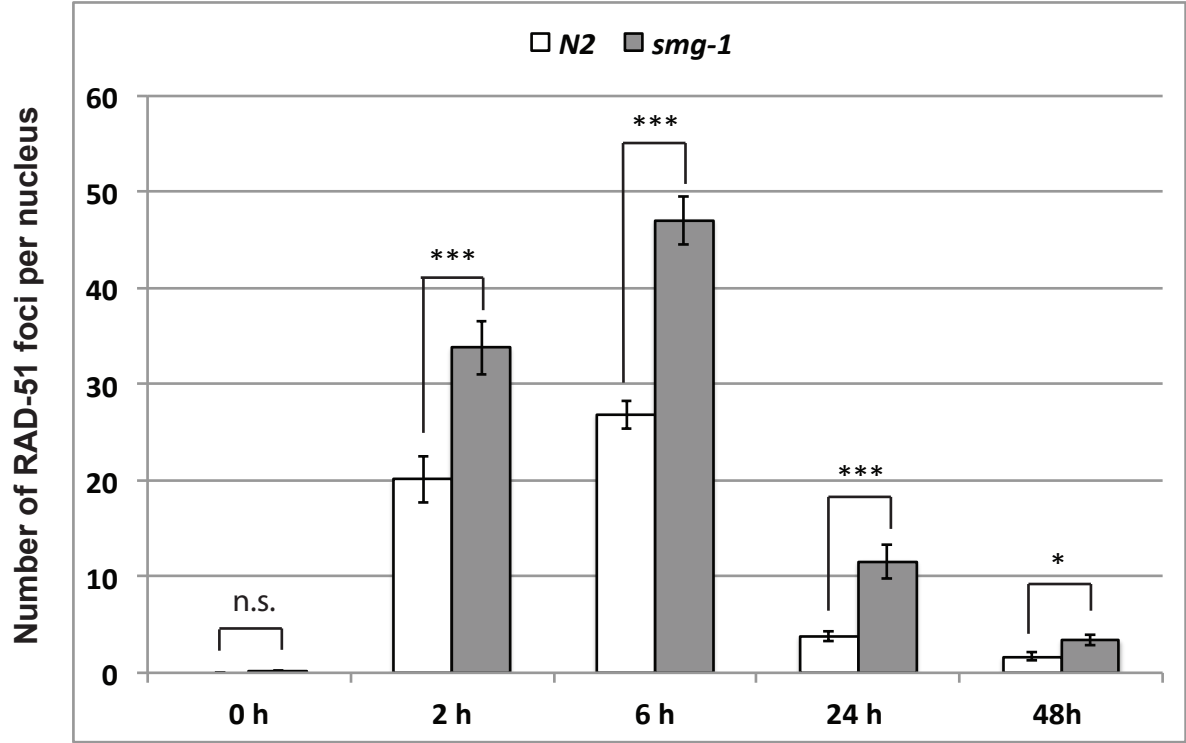

Supplement: Supplementary file 15 [file 1853FigureS4.pdf]

FIGURE S5

A

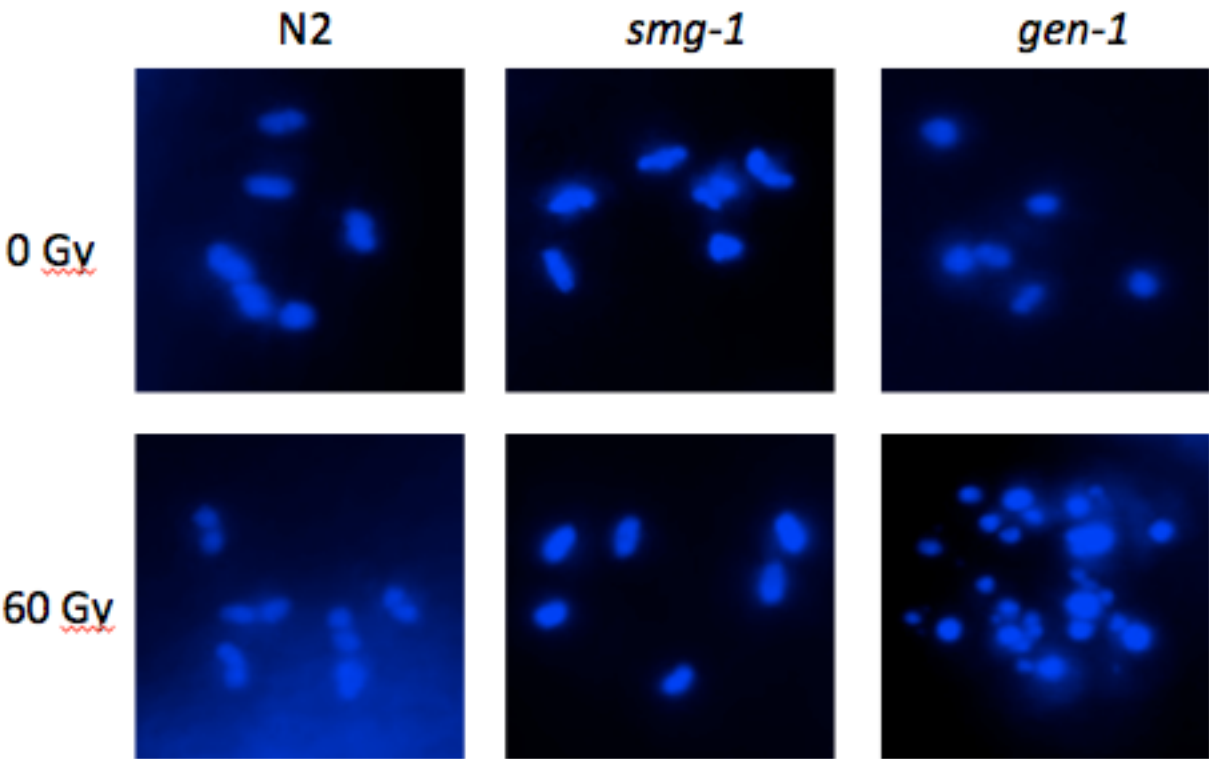

B

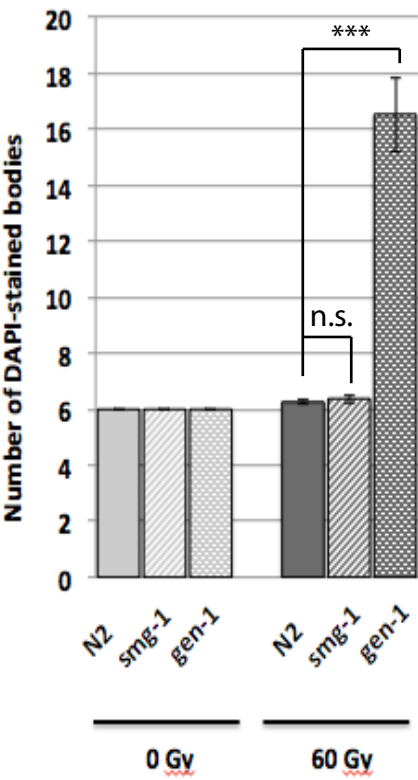

Supplement: Supplementary file 16 [file 1853FigureS5.pdf]
