# Supplementary material for: A Role for the Nonsense-Mediated mRNA Decay Pathway in Maintaining Genome Stability in Caenorhabditis elegans
Source: Genetics. 2017 Jun 20;206(4):1853–64. doi: 10.1534/genetics.117.203414 (PMC5560793; doi:10.1534/genetics.117.203414)
Supplement: Supplementary file 17 [file 1853TableS1.pdf]

Supplementary material

TABLE S1

Strain code	Genotype
TG1813	*N2 wt (Bristol)*
CB4856	*wt (Hawaiian)*
VC40686	*smg-1 (gk761853) I*
TR1331	*smg-1 (r861) I*
TG3855	*smg-1 (tg3855) I*
VC40212	*smg-5 (r860) I*
TG1660	*xpf-1 (tm2842) II*
TR2264	*smg-6 (ok1794) III*
DW102	*brc-1 (tm1145) III*
RB873	*lig-4 (ok716) III*
TG2534	*polq-1 (tm2026) III*
RB2422	*polh-1 (ok3317) III*
TG1540	*gen-1 (tm2940) III*
TG4211	*smg-1 (gk761853) I; brc-1 (tm1145) III*
TG4212	*smg-1 (gk761853) I; lig-4 (ok716) III*
TG4213	*smg-1 (gk761853) I; polq-1 (tm2026) III*
VC40827	*smg-1 (r904) I; unc-54 (r293) I*
TR1417	*smg-2 (r908) I; unc-54 (r293) I*
TR1335	*smg-5 (r860) I; unc-54 (r293) I*
TR1421	*unc-54 (r293) I; smg-3 (r930) IV*
TR1696	*unc-54 (r293) I; smg-4 (r1169) V*
TR1324	*unc-54 (r293) I; smg-6 (r1217) III*
TR2230	*unc-54 (r293) I; smg-7 (r1197) IV*
